# 2-[(4-Chloro­phen­yl)imino]-1,2-di­phenyl­ethanone

**DOI:** 10.1107/S2414314623000652

**Published:** 2023-01-31

**Authors:** Nouara Ziani, Brihi Ouarda, Soumia Kadri, Erwann Jeanneau, Ismail Warad, Amel Djedouani

**Affiliations:** a Département de Chimie, Faculté des Sciences, Université de Setif-1, El Bez, Setif, Algeria; b Laboratoire de Cristallographie, Département de Physique, Université des Frères Mentouri de Constantine-1, 25000 Constantine, Algeria; c Unité de Recherche de Chimie de l’Environnement, et Moléculaire Structurale (URCHEMS), Département de Chimie, Université des Frères Mentouri de Constantine-1, 25000 Constantine, Algeria; d Université de Lyon, Centre de Diffractométrie, Henri Longchambon, Villeurbanne, France; e Department of Chemistry, Science College, An-Najah National University, Nablus PO Box 7, Palestinian Territories; f Laboratoire de Physicochimie Analytique et de Cristallochimie, de Matériaux Organo-métalique et Biomoléculaire, 25000 Constantine, Algeria; g Ecole Normale Supérieure de Constantine, Université Constantine 3, 25000, Algeria; University of Aberdeen, United Kingdom

**Keywords:** crystal structure, benzil, C—H⋯O hydrogen bonds, Hirshfeld surface analysis

## Abstract

The title Schiff base, C_20_H_14_ClNO, obtained from the reaction of 4-chloro aniline with benzil, has an approximate T shape. The dihedral angle between the phenyl rings of the benzil unit is 74.14 (15)°. The extended structure features C—H⋯O hydrogen bonds.

## Abstract 2

In the title Schiff base, the dihedral angle between the phenyl rings of the benzil unit is 74.14 (5)°.

## Graphical abstract

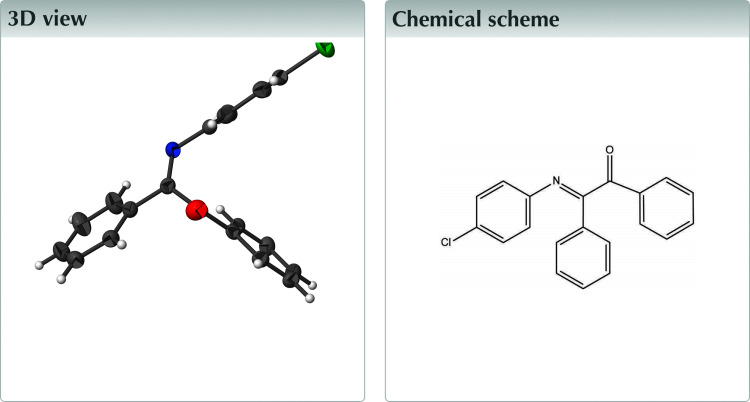

## Structure description

**Figure 1:**
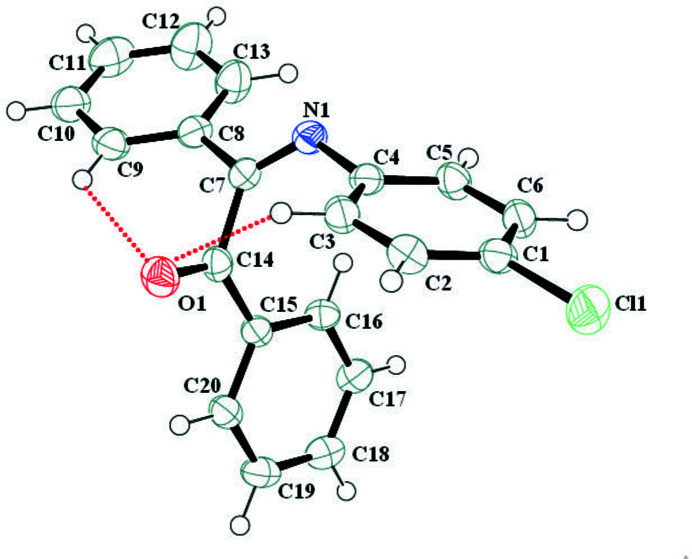
The title mol­ecule with the labelling scheme and 50% probability ellipsoids. Dashed lines indicate the intra­molecular hydrogen bonds.

There are only a few reported crystal structures of Schiff bases derived from benzil (Tabbiche *et al.*, 2022 ▸; Bouchama *et al.*, 2007 ▸; Bai *et al.*, 2006 ▸). We recently synthesized the title compound and we now report its crystal structure. The asymmetric unit contains one independent mol­ecule (Fig. 1 ▸). The O and the imine N atoms are *trans* with respect to the C7—C14 bond. The C1–C6 phenyl ring makes dihedral angles of 20.56 (6) and 74.03 (6)°with the C9–C10 and C15–C16 phenyl ring, respectively, of the benzil unit. The dihedral angle between the phenyl rings of the benzil unit is 74.14 (5)°. The C—N iminium bond length [1.268 (3) Å] is comparable to that observed in (*E*)-1-[4-({4-[(4-meth­oxy­benzyl­idene)amino]­phen­yl}sulfan­yl)phen­yl]ethan-1-one [1.252 (4) Å; Hebbachi *et al.*, 2015 ▸]. Atom O1 accepts two long and presumably weak intra­molecular hydrogen bonds with atoms H3 and H9 (Fig. 1 ▸), which generate *S*(6) and *S*(7) rings motifs, respectively: the former is approximately planar.

**Figure 2:**
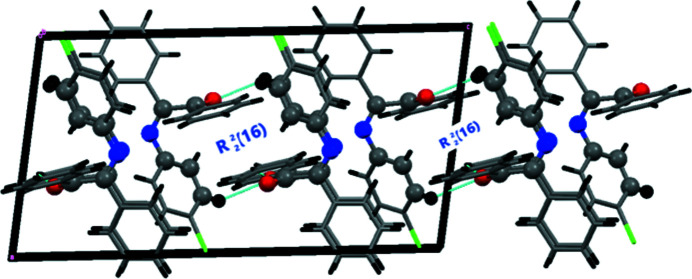
Packing arrangement of the title compound viewed along the *c-*axis direction. C— H⋯O hydrogen bonds are shown as dashed lines.

**Figure 3:**
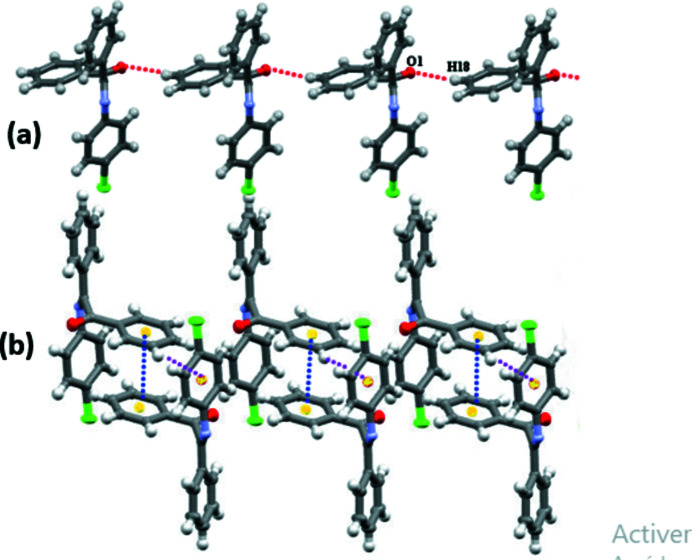
(*a*) View of part of the crystal structure, showing the formation of a hydrogen-bonded C18—H18⋯O1 chain and (*b*) the inter­molecular C—H⋯π(ring) and π–π stacking inter­actions bonds (violet and blue dashed lines, respectively) in the *ab* plane.

In the crystal, the mol­ecules are aligned head-to-foot along the *b-*axis direction, forming layers that extend in zigzag parallel to the *ac* plane. In the extended structure, two weak C—H⋯O hydrogen bonds help to consolidate the packing (Table 1 ▸, Fig. 2 ▸). The C18—H18⋯O1 hydrogen bonds generate a succession of infinite chains [graph set 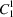 (7)] while C2—H2⋯O1 hydrogen bonds link the chains into layers, which are formed by a succession of 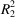 (16) rings, parallel to the *bc* plane [Fig. 3 ▸(*a*)]. Together, these hydrogen bonds lead to the formation of a three-dimensional network. Aromatic π–π stacking generates inversion dimers featuring the C15–C20 phenyl rings with a centroid–centroid distance of 3.744 (3) Å [Fig. 3 ▸(*b*)]. Along the *c-*axis direction, weak C—H⋯π(ring) inter­actions occur.

**Figure 4:**
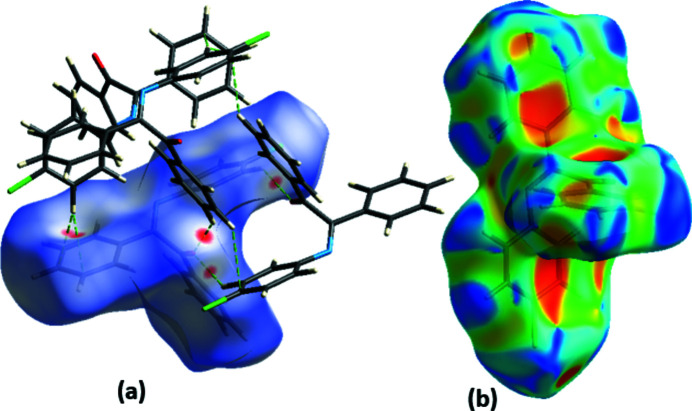
HS mapped over (*a*) *d*_norm_, showing the C—H⋯O and C—H⋯π inter­actions, and (*b*) shape-index.

A Hirshfeld surface (HS) analysis was performed and the associated two-dimensional fingerprint (FP) plots (Spackman & Jayatilaka, 2009 ▸) were generated using *Crystal Explorer 3.1* (Turner *et al.*, 2017 ▸). Fig. 4 ▸ shows the HS mapped over *d*_norm_ (–0.11 to 1.54 a.u.) and shape-index. The red spots in Fig. 4 ▸(*a*) reflect the formation of C—H⋯O, C—H⋯π and π–π stacking inter­actions. In the shape-index map [Fig. 4 ▸(*b*)], the adjacent red and blue triangle-like patches represent concave regions that indicate C—H⋯π(ring) and π–π stacking inter­actions. The two-dimensional FP plots indicate that the most important contributions to the packing, in descending percentage contribution, are from H⋯C (37.7%), H⋯H (34.6%), H⋯Cl (14.0%), H⋯O (6.1%), H⋯N (4.0%) and C⋯C (1.9%) contacts.

## Synthesis and crystallization

To a solution of benzil (2.1 g, 0.01 mmol) and 1 ml of acetic acid in ethanol (20 ml) was added 4-chloro aniline (0.01 mmol) dissolved in ethanol (15 ml). The mixture was stirred for 3 h under reflux. The product was isolated, recrystallized from ethanol solution and then dried in a vacuum to give the title compound (yield 59%; m.p. > 260°C). Yellow single crystals suitable for X-ray analysis were obtained by slow evaporation of a ethanol solution. IR ν, cm^−1^: 1594 (C=N, imine), 1660 (C=O), 3064 (aromatic C—H), 1212 (C—N) and 718 (C—Cl).

## Refinement

Crystal data, data collection and structure refinement details are summarized in Table 2 ▸.

## Supplementary Material

Crystal structure: contains datablock(s) I. DOI: 10.1107/S2414314623000652/hb4414sup1.cif

Structure factors: contains datablock(s) I. DOI: 10.1107/S2414314623000652/hb4414Isup2.hkl

Supporting information file. DOI: 10.1107/S2414314623000652/hb4414Isup3.cml

CCDC reference: 2237868

Additional supporting information:  crystallographic information; 3D view; checkCIF report

## Figures and Tables

**Table 1:** Hydrogen-bond geometry (Å, °) *Cg*1 is the centroid of the C1–C6 ring.

*D*—H⋯*A*	*D*—H	H⋯*A*	*D*⋯*A*	*D*—H⋯*A*
C3—H3⋯O1	0.93	2.67	3.247 (3)	120
C9—H9⋯O1	0.93	2.64	3.231 (3)	122
C2—H2⋯O1^i^	0.93	2.60	3.360 (3)	139
C19—H19⋯*Cg*1^ii^	0.93	2.88	3.689 (3)	146

Symmetry codes: (i) 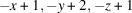 ; (ii) 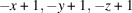 .

**Table 2:** Experimental details

Crystal data	
Chemical formula	C_20_H_14_ClNO
*M*_r_	320.78
Crystal system, space group	Monoclinic, *P*2_1_/*c*
Temperature (K)	293
*a*, *b*, *c* (Å)	10.0982 (12), 8.2447 (11), 19.365 (3)
β (°)	98.592 (12)
*V* (Å^3^)	1594.2 (4)
*Z*	4
Radiation type	Mo *K*α
μ (mm^−1^)	0.24
Crystal size (mm)	0.20 × 0.17 × 0.12

Data collection	
Diffractometer	Xcalibur, Atlas, Gemini ultra
Absorption correction	Analytical (*CrysAlis PRO*; Rigaku OD, 2018 ▸)
*T*_min_, *T*_max_	0.968, 0.974
No. of measured, independent and observed [*I* > 2σ(*I*)] reflections	13558, 4026, 2805
*R*_int_	0.045
(sin θ/λ)_max_ (Å^−1^)	0.700

Refinement	
*R*[*F* ^2^ > 2σ(*F* ^2^)], *wR*(*F* ^2^), *S*	0.062, 0.186, 1.11
No. of reflections	4026
No. of parameters	209
H-atom treatment	H-atom parameters constrained
Δρ_max_, Δρ_min_ (e Å^−3^)	0.38, −0.58

Computer programs: *CrysAlis PRO* (Rigaku OD, 2018 ▸), *SHELXT* (Sheldrick, 2015*a* ▸), *SHELXL* (Sheldrick, 2015*b* ▸) and *OLEX2* (Dolomanov *et al.*, 2009 ▸).

## full crystallographic data

### Crystal data

**Table:**

C_20_H_14_ClNO	*F*(000) = 668
*M**_r_* = 320.78	*D*_x_ = 1.337 Mg m^−^^3^
Monoclinic, *P*2_1_/*c*	Mo *K*α radiation, λ = 0.71073 Å
*a* = 10.0982 (12) Å	Cell parameters from 3987 reflections
*b* = 8.2447 (11) Å	θ = 4.3–29.1°
*c* = 19.365 (3) Å	µ = 0.24 mm^−^^1^
β = 98.592 (12)°	*T* = 293 K
*V* = 1594.2 (4) Å^3^	Block, clear pinkish yellow
*Z* = 4	0.20 × 0.17 × 0.12 mm

### Data collection

**Table:**

Xcalibur, Atlas, Gemini ultra diffractometer	2805 reflections with *I* > 2σ(*I*)
Detector resolution: 10.4685 pixels mm^-1^	*R*_int_ = 0.045
ω scans	θ_max_ = 29.9°, θ_min_ = 2.7°
Absorption correction: analytical (CrysAlisPro; Rigaku OD, 2018)	*h* = −13→14
*T*_min_ = 0.968, *T*_max_ = 0.974	*k* = −11→11
13558 measured reflections	*l* = −23→26
4026 independent reflections	

### Refinement

**Table:**

Refinement on *F*^2^	Hydrogen site location: inferred from neighbouring sites
Least-squares matrix: full	H-atom parameters constrained
*R*[*F*^2^ > 2σ(*F*^2^)] = 0.062	*w* = 1/[σ^2^(*F*_o_^2^) + (0.0695*P*)^2^ + 1.2547*P*] where *P* = (*F*_o_^2^ + 2*F*_c_^2^)/3
*wR*(*F*^2^) = 0.186	(Δ/σ)_max_ < 0.001
*S* = 1.11	Δρ_max_ = 0.38 e Å^−^^3^
4026 reflections	Δρ_min_ = −0.58 e Å^−^^3^
209 parameters	Extinction correction: SHELXL-2018/3 (Sheldrick 2015b), Fc^*^=kFc[1+0.001xFc^2^λ^3^/sin(2θ)]^-1/4^
0 restraints	Extinction coefficient: 0.0115 (19)

### Special details

**Table:**

Geometry. All esds (except the esd in the dihedral angle between two l.s. planes) are estimated using the full covariance matrix. The cell esds are taken into account individually in the estimation of esds in distances, angles and torsion angles; correlations between esds in cell parameters are only used when they are defined by crystal symmetry. An approximate (isotropic) treatment of cell esds is used for estimating esds involving l.s. planes.
Refinement. H-atom treatment: Fixed *U*_iso_ At 1.2 times of: All C(H) groups 2.a Aromatic/amide H refined with riding coordinates: C2(H2), C3(H3), C5(H5), C6(H6), C9(H9), C10(H10), C11(H11), C12(H12), C13(H13), C16(H16), C17(H17), C18(H18), C19(H19), C20(H20)

### Fractional atomic coordinates and isotropic or equivalent isotropic displacement parameters (Å^2^)

**Table:**

	*x*	*y*	*z*	*U*_iso_*/*U*_eq_	
C1	0.8370 (2)	0.8256 (3)	0.40271 (13)	0.0317 (5)	
C2	0.7484 (3)	0.9348 (3)	0.42442 (13)	0.0329 (5)	
H2	0.773837	0.997474	0.464122	0.040*	
C3	0.6214 (3)	0.9505 (3)	0.38673 (13)	0.0318 (5)	
H3	0.561128	1.023849	0.401135	0.038*	
C4	0.5837 (2)	0.8566 (3)	0.32725 (12)	0.0291 (5)	
C5	0.6759 (2)	0.7494 (3)	0.30579 (13)	0.0335 (6)	
H5	0.652069	0.688099	0.265528	0.040*	
C6	0.8023 (2)	0.7329 (3)	0.34361 (13)	0.0330 (5)	
H6	0.863287	0.660176	0.329374	0.040*	
C7	0.3486 (2)	0.8532 (3)	0.30665 (13)	0.0289 (5)	
C8	0.2203 (2)	0.8607 (3)	0.25828 (13)	0.0322 (5)	
C9	0.1038 (3)	0.9201 (4)	0.27901 (15)	0.0402 (6)	
H9	0.104682	0.955946	0.324621	0.048*	
C10	−0.0139 (3)	0.9263 (4)	0.23202 (17)	0.0480 (8)	
H10	−0.091026	0.969215	0.245795	0.058*	
C11	−0.0170 (3)	0.8691 (4)	0.16500 (17)	0.0521 (8)	
H11	−0.096307	0.872138	0.133672	0.062*	
C12	0.0979 (3)	0.8075 (4)	0.14449 (18)	0.0547 (8)	
H12	0.095682	0.767683	0.099394	0.066*	
C13	0.2163 (3)	0.8044 (4)	0.19061 (15)	0.0458 (7)	
H13	0.293727	0.764354	0.176137	0.055*	
C14	0.3371 (2)	0.8150 (3)	0.38222 (12)	0.0292 (5)	
C15	0.3471 (2)	0.6436 (3)	0.40449 (12)	0.0268 (5)	
C16	0.3846 (2)	0.5223 (3)	0.36134 (12)	0.0306 (5)	
H16	0.403267	0.548117	0.317048	0.037*	
C17	0.3940 (3)	0.3635 (3)	0.38435 (14)	0.0359 (6)	
H17	0.420029	0.282786	0.355635	0.043*	
C18	0.3652 (3)	0.3243 (3)	0.44943 (14)	0.0377 (6)	
H18	0.370854	0.216977	0.464414	0.045*	
C19	0.3277 (3)	0.4438 (3)	0.49268 (14)	0.0363 (6)	
H19	0.308420	0.416916	0.536746	0.044*	
C20	0.3191 (2)	0.6030 (3)	0.47047 (12)	0.0311 (5)	
H20	0.294378	0.683342	0.499741	0.037*	
Cl1	0.99595 (7)	0.80122 (10)	0.45011 (4)	0.0468 (3)	
N1	0.4585 (2)	0.8715 (3)	0.28342 (10)	0.0312 (5)	
O1	0.31698 (19)	0.9261 (2)	0.42112 (10)	0.0386 (5)	

### Atomic displacement parameters (Å^2^)

**Table:**

	*U*^11^	*U*^22^	*U*^33^	*U*^12^	*U*^13^	*U*^23^
C1	0.0287 (12)	0.0394 (13)	0.0268 (12)	−0.0027 (10)	0.0034 (9)	0.0037 (10)
C2	0.0341 (13)	0.0343 (13)	0.0306 (12)	−0.0052 (11)	0.0054 (10)	−0.0028 (10)
C3	0.0315 (12)	0.0310 (12)	0.0339 (13)	0.0012 (10)	0.0081 (10)	−0.0001 (10)
C4	0.0277 (12)	0.0333 (12)	0.0261 (11)	−0.0009 (10)	0.0036 (9)	0.0052 (9)
C5	0.0303 (12)	0.0443 (14)	0.0262 (12)	0.0002 (11)	0.0047 (10)	−0.0041 (10)
C6	0.0273 (12)	0.0423 (14)	0.0299 (12)	0.0022 (11)	0.0063 (10)	−0.0026 (10)
C7	0.0305 (12)	0.0253 (11)	0.0310 (12)	0.0026 (10)	0.0051 (10)	0.0018 (9)
C8	0.0312 (12)	0.0317 (12)	0.0329 (13)	−0.0005 (10)	0.0019 (10)	0.0048 (10)
C9	0.0323 (13)	0.0520 (16)	0.0369 (14)	0.0013 (12)	0.0077 (11)	0.0126 (12)
C10	0.0293 (13)	0.0615 (19)	0.0535 (18)	0.0031 (13)	0.0069 (13)	0.0206 (15)
C11	0.0379 (16)	0.0588 (19)	0.0537 (19)	−0.0061 (14)	−0.0123 (14)	0.0087 (15)
C12	0.0476 (18)	0.064 (2)	0.0466 (18)	0.0059 (15)	−0.0120 (14)	−0.0122 (15)
C13	0.0417 (16)	0.0536 (17)	0.0396 (16)	0.0099 (13)	−0.0026 (12)	−0.0083 (13)
C14	0.0240 (11)	0.0337 (12)	0.0298 (12)	0.0020 (10)	0.0040 (9)	−0.0024 (9)
C15	0.0231 (11)	0.0344 (12)	0.0222 (11)	−0.0005 (9)	0.0007 (8)	−0.0010 (9)
C16	0.0338 (13)	0.0320 (12)	0.0253 (11)	−0.0010 (10)	0.0022 (9)	0.0002 (9)
C17	0.0425 (15)	0.0315 (12)	0.0318 (13)	0.0036 (11)	−0.0007 (11)	−0.0031 (10)
C18	0.0384 (14)	0.0354 (13)	0.0372 (14)	−0.0040 (11)	−0.0010 (11)	0.0063 (11)
C19	0.0353 (13)	0.0434 (15)	0.0296 (13)	−0.0062 (11)	0.0036 (10)	0.0081 (10)
C20	0.0302 (12)	0.0364 (13)	0.0274 (12)	−0.0008 (10)	0.0060 (10)	−0.0010 (9)
Cl1	0.0312 (4)	0.0681 (5)	0.0381 (4)	0.0023 (3)	−0.0047 (3)	−0.0040 (3)
N1	0.0274 (10)	0.0365 (11)	0.0290 (10)	0.0012 (9)	0.0022 (8)	0.0043 (8)
O1	0.0460 (11)	0.0336 (9)	0.0378 (10)	0.0018 (8)	0.0115 (8)	−0.0051 (8)

### Geometric parameters (Å, º)

**Table:**

C1—C6	1.377 (4)	C10—H10	0.9300
C1—C2	1.379 (4)	C11—C12	1.378 (5)
C1—Cl1	1.737 (3)	C11—H11	0.9300
C2—C3	1.384 (3)	C12—C13	1.382 (4)
C2—H2	0.9300	C12—H12	0.9300
C3—C4	1.393 (3)	C13—H13	0.9300
C3—H3	0.9300	C14—O1	1.222 (3)
C4—C5	1.392 (4)	C14—C15	1.477 (3)
C4—N1	1.419 (3)	C15—C20	1.390 (3)
C5—C6	1.380 (3)	C15—C16	1.391 (3)
C5—H5	0.9300	C16—C17	1.382 (4)
C6—H6	0.9300	C16—H16	0.9300
C7—N1	1.268 (3)	C17—C18	1.374 (4)
C7—C8	1.482 (3)	C17—H17	0.9300
C7—C14	1.518 (3)	C18—C19	1.382 (4)
C8—C13	1.385 (4)	C18—H18	0.9300
C8—C9	1.388 (4)	C19—C20	1.380 (4)
C9—C10	1.385 (4)	C19—H19	0.9300
C9—H9	0.9300	C20—H20	0.9300
C10—C11	1.377 (5)		
			
C6—C1—C2	121.3 (2)	C10—C11—H11	120.1
C6—C1—Cl1	118.4 (2)	C12—C11—H11	120.1
C2—C1—Cl1	120.3 (2)	C11—C12—C13	120.3 (3)
C1—C2—C3	119.5 (2)	C11—C12—H12	119.8
C1—C2—H2	120.2	C13—C12—H12	119.8
C3—C2—H2	120.2	C12—C13—C8	120.4 (3)
C2—C3—C4	120.1 (2)	C12—C13—H13	119.8
C2—C3—H3	120.0	C8—C13—H13	119.8
C4—C3—H3	120.0	O1—C14—C15	123.2 (2)
C5—C4—C3	119.2 (2)	O1—C14—C7	118.9 (2)
C5—C4—N1	116.9 (2)	C15—C14—C7	117.9 (2)
C3—C4—N1	123.7 (2)	C20—C15—C16	119.3 (2)
C6—C5—C4	120.7 (2)	C20—C15—C14	119.0 (2)
C6—C5—H5	119.6	C16—C15—C14	121.7 (2)
C4—C5—H5	119.6	C17—C16—C15	119.9 (2)
C1—C6—C5	119.1 (2)	C17—C16—H16	120.0
C1—C6—H6	120.4	C15—C16—H16	120.0
C5—C6—H6	120.4	C18—C17—C16	120.3 (2)
N1—C7—C8	120.0 (2)	C18—C17—H17	119.8
N1—C7—C14	124.3 (2)	C16—C17—H17	119.8
C8—C7—C14	115.6 (2)	C17—C18—C19	120.2 (2)
C13—C8—C9	119.1 (2)	C17—C18—H18	119.9
C13—C8—C7	118.9 (2)	C19—C18—H18	119.9
C9—C8—C7	122.0 (2)	C20—C19—C18	120.0 (2)
C10—C9—C8	120.3 (3)	C20—C19—H19	120.0
C10—C9—H9	119.9	C18—C19—H19	120.0
C8—C9—H9	119.9	C19—C20—C15	120.2 (2)
C11—C10—C9	120.2 (3)	C19—C20—H20	119.9
C11—C10—H10	119.9	C15—C20—H20	119.9
C9—C10—H10	119.9	C7—N1—C4	121.8 (2)
C10—C11—C12	119.8 (3)		

### Hydrogen-bond geometry (Å, º)

*Cg*1 is the centroid of the C1–C6 ring.

**Table:**

*D*—H···*A*	*D*—H	H···*A*	*D*···*A*	*D*—H···*A*
C3—H3···O1	0.93	2.67	3.247 (3)	120
C9—H9···O1	0.93	2.64	3.231 (3)	122
C2—H2···O1^i^	0.93	2.60	3.360 (3)	139
C19—H19···*Cg*1^ii^	0.93	2.88	3.689 (3)	146

Symmetry codes: (i) −*x*+1, −*y*+2, −*z*+1; (ii) −*x*+1, −*y*+1, −*z*+1.
